# Validation of Nova Stat Profile Prime Plus point-of-care testing for dialysis in South Africa

**DOI:** 10.4102/ajlm.v14i1.2663

**Published:** 2025-07-08

**Authors:** Kagiso M. Masemola, Ngalulawa Kone, Chemedzai Chikomba, Siyabonga Khoza

**Affiliations:** 1 Department of Chemical Pathology, Faculty of Health Sciences, University of the Witwatersrand, Johannesburg, South Africa; 2 Department of Chemical Pathology, National Health Laboratory Service, Johannesburg, South Africa

**Keywords:** creatinine index, critical care analysers, haemodialysis adequacy, Nova Stat Profile Prime Plus, point-of-care testing

## Abstract

**Background:**

Chronic kidney disease is a global health crisis, and delays in laboratory testing worsen outcomes. Point-of-care testing (POCT) has shown utility in various settings, but its performance at high creatinine levels seen in advanced chronic kidney disease is variable.

**Objective:**

We evaluated the analytical and clinical performance of the Nova Stat Profile Prime Plus (SPP+) point-of-care analyser among patients on maintenance haemodialysis.

**Methods:**

A prospective study was conducted at Chris Hani Baragwanath Hospital, Johannesburg, South Africa. In phase one (01 June 2023 – 31 July 2023), precision, linearity, and accuracy of SPP+ were assessed using remnant patient samples. Blood gases, electrolytes, and metabolic parameters were compared with the GEM Premier 5000, while urea and creatinine were compared with the Roche cobas c702. In phase two (01 November 2023 – 31 December 2023), SPP+ was clinically validated among adults undergoing haemodialysis. Whole blood was collected pre- and post-dialysis to assess dialysis adequacy and the creatinine index.

**Results:**

In phase one, SPP+ showed acceptable precision (coefficients of variation 0% – 2.7%), linearity, and correlation (*r* > 0.90). Creatinine showed proportional bias (4.56% [0.33 – 8.80]) at higher concentrations. Among 51 haemodialysis patients (22 women, 29 men; aged 32–51 years), SPP+ showed 88.6% agreement for single pool Kt/V and urea reduction ratio. However, creatinine index agreement was low (34.3%, Cohen’s kappa = 0.24, *p* < 0.0001).

**Conclusion:**

Nova SPP+ was comparable to central laboratory analysis, though caution is needed at high creatinine levels, where central laboratory analysis remains the preferred choice.

**What this study adds:**

This study provides the first South African data on POCT in haemodialysis. The analyser has demonstrated potential for monitoring, but performance concerns remain at high creatinine levels. The findings offer practical guidance for integrating POCT into advanced chronic kidney disease care in a resource-limited setting.

## Introduction

Point-of-care testing (POCT) on blood gas analysers has evolved from solely measuring pH and blood gases to sophisticated analysers capable of assessing electrolytes and metabolic parameters.^1^ Point-of-care testing enables testing closer to patients by decentralising certain laboratory diagnostic services.^2^ It provides rapid access to laboratory test results for clinical decision-making and potential for improved outcomes. However, despite these advantages, when managed by non-laboratory staff, POCT is not error-proof, and can be susceptible to both preanalytical, analytical as well as post-analytical errors.^2,3^

The new ISO 15189:2022, incorporating ISO 15189:2012 and ISO 22870:2016, mandates appointing trained individuals for POCT quality.^4^ Laboratory experts play a crucial role in overseeing quality, offering expertise in device selection, user training, quality assurance, risk reduction and management of the total testing process.^5^ Comprehensive method verifications, such as health technology assessments, assist in establishing POCT device performance and evaluating its clinical utility.^6^ While there is no consensus on the scope of method verification, Clinical and Laboratory Standards Institute (CLSI) guidelines offer a structured framework for evaluating analytical performance.^5^

Nova Biomedical (Nova Biomedical, Waltham, Massachusetts, United States) has incorporated advancements in POCT into the Nova Stat Profile Prime Plus (SPP+) analyser, utilising microsensor technology with capability to measure pH, partial pressure of carbon dioxide, partial pressure of oxygen, sodium, potassium, chloride, ionised calcium, ionised magnesium, glucose, lactate, creatinine, urea and total bilirubin. A South African study in 2020 evaluated that the SPP+ demonstrated acceptable precision and correlation with other POCT analysers.^7^ While literature supports SPP+ as an analyser yielding satisfactory results across diverse clinical settings,^8,9,10^ a 2018 study in the United Kingdom emphasises the need for caution in interpreting results, particularly in the context of screening for post-contrast acute kidney injury.^8^ This caution is warranted, because of the low level of agreement of the SPP+ in comparison with other similar analysers.^9^ This finding is supported by a 2023 study in Nicaragua and Guatemala which showed that, as concentrations of measured creatinine increase in chronic kidney disease (CKD), point-of-care creatinine is less accurate.^10^ However, this has not been evaluated with the SPP+. Therefore, robust analytical and clinical verification is needed before considering broader implementation of this POCT analyser.

Treatment for patients with advanced CKD through haemodialysis aims to improve quality of life, while also extending life expectancy as an additional goal. However, achieving haemodialysis adequacy does not equate to adequate patient care.^11^ Haemodialysis-dependent patients require treatments beyond haemodialysis alone. This is reflected in the Kidney Disease Outcomes Quality Initiative guidelines, which cover various aspects of care such as anaemia, nutrition, metabolic bone disease, diabetes, and cardiovascular disease.^12^ As a priority, the Kidney Disease Outcomes Quality Initiative recommends assessing haemodialysis adequacy through formal urea kinetic modeling as a proxy for small solute clearance, specifically using single-pool Kt/V (spKt/V).^11,13^ This calculation involves the dialyser’s urea clearance rate (K), haemodialysis hours (t), and total body water content represented by the urea distribution volume (V).^11^ For thrice-weekly schedules, adequate haemodialysis requires a minimum spKt/V of 1.2, with an optimal target of 1.4 or, in resource-limited settings, a urea reduction ratio (URR) of 65% per session.^11,13^

While small solute clearance is a priority, assessing dialysis adequacy should also consider other important outcomes in this population.^11^ Protein-energy wasting (PEW) is prevalent in advanced CKD and highest in patients with kidney failure.^12,14,15^ This is because PEW results from factors other than reduced nutrient intake, such as catabolic illness, oxidative stress, or loss of biological substances in urine and dialysate.^15,16^ Several studies emphasise PEW’s prognostic significance as a strong predictor of morbidity and mortality, irrespective of haemodialysis adequacy. The Kidney Disease Outcomes Quality Initiative guidelines recommend using creatinine kinetics to estimate muscle mass and, by extension, PEW in adults with kidney failure.^11^ A simple tool is the creatinine index (C_index_) reflecting dietary protein intake and skeletal muscle mass, with a reduced C_index_ (< 22 mg/kg/day) linked to poor patient survival.^17^

Frequent assessments of haemodialysis adequacy are recommended,^12^ but the centralised laboratory system in South Africa poses practical challenges.^18^ In this system, same-day test results are often unavailable because of pre-analytical challenges such as sample transportation and excessive laboratory workload. In response to these delays, healthcare professionals advise patients to schedule their appointments for a later time, thereby prolonging diagnostic turnaround times.^3,18,19^ Measurements of urea and creatinine on blood gas analysers offer a potential solution, with demonstrated comparability to central laboratory analysers.^20^ However, no studies have evaluated the performance of SPP+ for haemodialysis adequacy or PEW. Therefore, the aim of this study was to evaluate both the analytical performance of the SPP+ blood gas analyser and its clinical performance in haemodialysis.

## Methods

### Ethical considerations

The study received ethical clearance from the University of the Witwatersrand Human Research Ethics Committee. Approval for the use of remnant patient samples submitted for routine diagnostic testing was granted under clearance number M2111171, with the requirement for patient consent waived by the committee. For the prospective phase involving recruited participants, separate approval was obtained under clearance number M230207, and written informed consent was obtained prior to participation. Demographic data were collected using de-identified questionnaires. All responses were recorded anonymously, and stored data were protected with password-secured access.

### Study design

A prospective, observational study was conducted from 01 June 2023 to 31 December 2023, at the National Health Laboratory Services, which is affiliated with Chris Hani Baragwanath Academic Hospital located in Johannesburg, South Africa. This facility is a tertiary healthcare institution.

### Participants

The study was conducted in two phases: The first phase evaluated the SPP+ analyser’s precision,^21^ linearity^22^ and accuracy^23^ using CLSI protocols with quality control material, external quality assessment materials, and remnant patient samples. This phase was conducted from 01 June 2023 to 31 July 2023.

The second phase (clinical validation) involved consecutive recruitment of participants from the adult chronic haemodialysis unit at Chris Hani Baragwanath Academic Hospital between 01 November 2023 and 31 December 2023. This unit caters for 72 patients weekly, all undergoing thrice-weekly haemodialysis. Participants who did not complete a haemodialysis session for any reason, and those on acute dialysis, were excluded. Individuals who agreed to participate provided two 5 mL whole blood samples before and after a haemodialysis session.

Phase two participants completed a questionnaire to collect information required for calculation of haemodialysis adequacy and C_index_, including age, sex, and pre- and post-haemodialysis weight. The questionnaire was created by the authors and submitted to the ethics committee. It was administered by the primary researcher through an interview to ensure that patients understood the form. Responses were recorded on paper forms.

### Sample collection and analysis

#### Phase one: Analytical verification

**Precision study (CLSI EP15):** Within-run and between-run imprecision were evaluated using Stat Profile Prime Plus^®^ Blood Gas/CO-Oximeter controls 1, 2, and 3, as well as chemistry controls 4 and 5 from Nova Biomedical. Five-replicate runs of each control level were run daily for five consecutive days.^21^

**Linearity study (CLSI EP06):** Linearity across the measuring range was assessed utilising at least five concentration levels. For electrolytes and metabolic parameters, Nova Linearity levels 1–4 were analysed. Because of the instability of blood gas analytes, external quality assessment materials from Bio-Rad External Quality Assurance Services and the National Health Laboratory Service proficiency schemes were used as additional concentration levels. These materials were lyophilised and reconstituted prior to analysis. Linearity materials were analysed in triplicate.^22^

**Method comparison (CLSI EP09):** Forty-five whole blood (sodium-heparin) remnant samples from in-hospital patients were used to compare the analyser to currently used routine analysers in our laboratory, namely GEM Premier 5000 blood gas analyser (Werfen S.A., Barcelona, Spain) for blood gas and electrolyte tests, and the Roche cobas 8000 c702 analyser (Roche Diagnostics, Rotkreuz, Switzerland) for urea and creatinine.^23^

Potentiometric sensors were used for analysis of pH, partial pressure of carbon dioxide, sodium, potassium, chloride, and ionised calcium on SPP+ and GEM Premier 5000 (Online Supplementary Table 1). Amperometry was used to evaluate partial pressure of oxygen, glucose and lactate. Urea and creatinine were measured using coupled enzymatic reactions with electrochemical detection. On the Roche cobas c702 module, urea and creatinine were measured using enzymatic principles coupled with spectrophotometric detection.

#### Phase two: Clinical validation

During the pre-haemodialysis step, 5 mL of peripheral blood was drawn into lithium-heparin syringes by the unit nursing staff. Then, using the stop-dialysate-flow technique, nursing staff promptly collected an additional 5 mL of blood from the dialyser input port in the post-haemodialysis step.

The whole blood samples were first analysed on the SPP+ and thereafter aliquoted into 1 mL microcentrifuge tubes and centrifuged at 3500 rpm for 10 min. The plasma was then transferred into 3 mL Hitachi cups for analysis on the Roche cobas c702 module.

### Statistical analysis

Results were captured on Microsoft Excel (Version 2312, Microsoft Corporation, Redmond, Washington, United States). Biochemical results were extracted from the analysers and questionnaire responses initially recorded on paper were entered manually into Excel using single data entry which was later verified.

#### Analytical verification

Statistical tests were performed following CLSI protocols.^21,22,23^

The National Health Laboratory Services EP15 Excel template adapted from the CLSI, EP evaluator (Version 12.3.0.2, Clinical and Laboratory Standards Institute, Wayne, Pennsylvania, United States) and MedCalc^®^ Statistical Software (Version 22.009, MedCalc Software Ltd., Ostend, Belgium) were used for statistical analysis.

Analytical performance specifications (APS) were chosen based on one of three models from the Milan conference.^2^ Biological variation data were obtained from the European Federation of Clinical Chemistry and Laboratory Medicine and Westgard databases. When the latest biological variation data for an analyte were unavailable, we used the total allowable error limits from the Royal College of Pathologists of Australasia and the Clinical Laboratory Improvement Amendments.

**Precision (CLSI EP15):** Mean concentration, standard deviation (s.d.), coefficient of variation, and bias were calculated using the one-way analysis of variance test. The manufacturer’s performance claims (Online Supplementary Table 2) and APS (Online Supplementary Table 3) were used for acceptance criteria.

**Linearity study (CLSI EP06):** Using EP Evaluator (12.3.0.2), linearity was acceptable if the deviation from the unweighted regression line was within the allowable deviation limits for each analyte.

**Method comparison (CLSI EP09):** Tukey’s rule was used to test for outliers. Passing-Bablok regression was used to show the correlation between methods. Bland-Altman plots were used to define method agreement intervals, and these were compared to total allowable error limits for each analyte.

#### Clinical validation

Haemodialysis adequacy was assessed using SpKt/V and URR. SpKt/V was calculated via the second-generation Daugirdas equation^11^ (Equation 1):


$$\begin{array}{l} \frac{SpKt}{V}=-\ln\left(\left(\frac{postHDurea}{PreHDurea}\right)-0.03\right)\\ \;+\left(4-3.5*\left(\frac{postHDurea}{PreHDurea}\right)\right)*(ultrafiltratevolume/weight) \end{array}$$ [Eqn 1]

Urea reduction ratio was calculated as a ratio^13^ (Equation 2):


$$URR=\left(\frac{preHDurea-postHDurea}{preHDurea}\right)*100\%$$ [Eqn 2]

Two cut-off points (a minimum spKt/V of 1.2 and an optimal spKt/V of 1.4) were used to assess the level of agreement between methods for haemodialysis adequacy.

Furthermore, participants were then classified into two groups based on haemodialysis adequacy targets determined by URR, with a minimum URR of 65% per session. Protein-energy wasting was estimated utilising the creatinine index (C_index_).^17^ Calculation of C_index_ involved the modified C_index_ equation, incorporating the participant’s age, sex, pre-haemodialysis creatinine concentration, and calculated spKt/V.^17^

The Shapiro-Wilk test was used to assess normality of spKt/V, URR and C_index_ results from both analysers. Statistical comparison was done using the Wilcoxon Signed Rank test; *p* < 0.05 was considered statistically significant. Inter-rater agreement was assessed with percentage agreement and the Cohen’s kappa statistic.

## Results

### Analytical verification

#### Precision

All analytes met manufacturer claims and APS criteria for within-run and between-run imprecision (Table 1).

**TABLE 1:** Assessment of within-run and between-run precision using Stat Profile Prime Plus ampouled controls (*N* = 25), Johannesburg, South Africa, June 2023 to July 2023.

Analyte	Quality control material	Quality control level	Target mean	Within-run CV? %	Between-run CV %	Performance
pH	Stat Profile Prime Plus ampouled controls	1	7.21	0.0	0.1	Acceptable
		2	7.43	0.0	0.0	
		3	7.62	0.0	0.1	
pCO_2_ (mmHg)	Stat Profile Prime Plus ampouled controls	1	60.5	1.5	2.1	Acceptable
		2	37.0	0.8	1.8	
		3	20.5	1.9	2.3	
pO_2_ (mmHg)	Stat Profile Prime Plus ampouled controls	1	63.5	2.3	2.7	Acceptable
		2	113.5	0.7	0.7	
		3	152.1	1.0	1.1	
Sodium (mmol/L)	Stat Profile Prime Plus ampouled controls	4	138.3	0.2	0.3	Acceptable
		5	111.6	0.2	0.4	
Potassium (mmol/L)	Stat Profile Prime Plus ampouled controls	4	3.98	0.1	0.1	Acceptable
		5	6.34	0.3	0.6	
Chloride (mmol/L)	Stat Profile Prime Plus ampouled controls	4	123.4	0.2	0.6	Acceptable
		5	96.6	0.3	0.5	
Ionised calcium (mmol/L)	Stat Profile Prime Plus ampouled controls	4	1.08	0.3	0.5	Acceptable
		5	1.58	0.6	1.2	
Glucose (mmol/L)	Stat Profile Prime Plus ampouled controls	4	4.4	0.9	0.9	Acceptable
		5	16.2	0.9	1.5	
Lactate (mmol/L)	Stat Profile Prime Plus ampouled controls	4	1.7	1.7	1.7	Acceptable
		5	6.5	1.4	2.0	
Urea (mmol/L)	Stat Profile Prime Plus ampouled controls	4	5.7	0.7	1.2	Acceptable
		5	18.6	0.6	0.9	
Creatinine (μmol/L)	Stat Profile Prime Plus ampouled controls	4	97	1.0	1.3	Acceptable
		5	645	1.0	1.4	

CV, coefficient of variation; pCO_2_, partial pressure of carbon dioxide; pO_2_, partial pressure of oxygen.

#### Method comparison

**Passing-Bablok analysis:** There was a good correlation between GEM Premier and SPP+ for all analytes (*r* > 0.90), except for sodium (*r* = 0.88) (Table 2).

**TABLE 2:** Summary of Passing-Bablok regression parameters for method comparison and analytical precision assessment Johannesburg, South Africa, June 2023 to July 2023.

Analyte	Intercept A	95% CI	Slope B	95% CI	Correlation coefficient†	95% CI
pH	0.080	−0.514–0.758	0.994	0.900–1.075	0.968	0.942–0.982
pCO_2_	−1.000	−1.000–3.900	1.000	0.900–1.000	0.978	0.960–0.988
pO_2_	0.839	0.000–2.338	0.989	0.969–1.000	0.994	0.989–0.997
Sodium (mmol/L)	−25.295	−56.790–0.600	1.190	1.000–1.420	0.876	0.788–0.929
Potassium (mmol/L)	0.450	0.291–0.611	0.900	0.861–0.938	0.984	0.972–0.991
Chloride (mmol/L)	10.520	0.843–19.817	0.920	0.833–1.013	0.904	0.830–0.947
Ionised Calcium	0.156	0.086–0.230	0.875	0.809–0.937	0.927	0.871–0.960
Glucose	0.100	−0.126–0.300	1.000	0.958–1.035	0.995	0.990–0.997
Lactate	−0.140	−0.241–0.002	1.080	1.032–1.118	0.993	0.987–0.996
Urea	0.559	0.435–0.685	0.853	0.819–0.882	0.995	0.991–0.997
Creatinine	15.041	9.289–20.494	0.796	0.737–0.844	0.958	0.924–0.977

CI, confidence interval; pCO_2_, partial pressure of carbon dioxide; pO_2_, partial pressure of oxygen.

† , *p* < 0.0001.

A comparison of SPP+ and GEM Premier 5000 revealed biases for sodium and chlorise. Sodium showed a constant negative bias (intercept of −25.3 [−56.79–0.60]). with a regression slope of 1.2. Chloride had a Spearman rank correlation coefficient of 0.90, but showed a positive constant bias (intercept of 10.52) with a confidence interval that did not include the ideal of zero (0.84–19.82).

**FIGURE 1:**
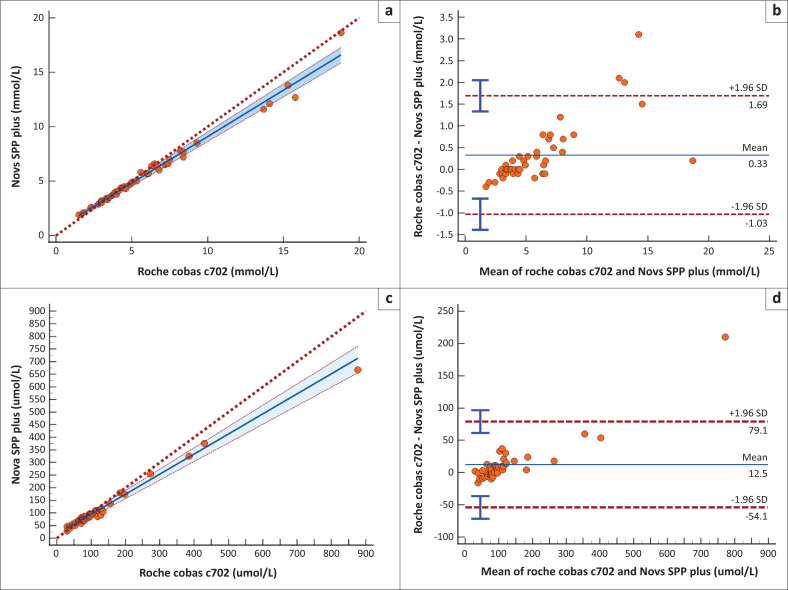
Correlation of SPP+ and Roche Cobas c702 results for urea and creatinine, Johannesburg, South Africa, June 2023 to July 2023. (a) Passing–Bablok regression analysis (*y* = 0.559 + 0.853*x*; *n* = 45) and (b) Bland–Altman plots for urea. Regression analysis displays the regression line (solid), confidence intervals (dashed), and identity line (*x* = *y*, dotted). (c) Passing–Bablok regression analysis (*y* = 15.041 + 0.796*x*; *n* = 45) and (d) Bland–Altman plots for creatinine. Bland-Altman plots depict differences versus the mean with mean (solid blue) and limits of agreement (dotted red) lines. 95% confidence intervals for the limits of agreement are indicated by blue error bars.

Comparison of urea and creatinine on SPP+ and Roche cobas c702 demonstrated an acceptable correlation (*r* > 0.95) for both analytes, but revealed systematic errors (Figure 1). Urea had a slope of 0.85 and a *y*-intercept of −0.56 when compared to plasma urea. Creatinine on SPP+ showed a positive constant error (*y*-intercept = 15.04) and a proportional error with a slope of 0.78. Although the method comparison study covered the reference interval for all analytes, it did not assess extremes of the measuring range due to the limited availability of lithium-heparin remnant samples.

**Bland-Altman plots:** On evaluation of method agreement, all analytes had percentage bias within the stipulated APS except chloride (Table 3). The average percentage difference for chloride between for SPP+ and GEM Premier 5000 was –2.2% which is more than the 0.6% minimum bias analytical performance specification by the European Federation of Clinical Chemistry and Laboratory Medicine and more than 1.5% minimum bias by the Royal College of Pathologists of Australasia (RCPA). However, the bias was within Clinical Laboratory Improvement Amendments limits of 2.5%.

**TABLE 3:** Bland–Altman plots for method comparison and agreement analysis using the Nova Stat Profile Prime Plus, Johannesburg, South Africa, June 2023 to July 2023.

Analyte	Measurement units	Bland-Altman plots					
		Bias	95% CI	Lower limit of agreement	95% CI	Upper limit of agreement	95% CI
pH	Absolute	−0.030	−0.037 to 0.024	−0.067	−0.077 to −0.0572	0.007	−0.003 to 0.0167
	%	0.411	0.488 to 0.333	−0.9134	−1.047 to −0.781	0.093	−0.039 to 0.225
pCO_2_	Absolute	1.178	0.629 to 1.726	−2.399	−3.344 to −1.455	4.755	3.811 to 5.699
	%	2.360	1.199 to 3.522	−5.218	−7.218 to −3.218	9.939	7.939 to 11.939
pO_2_	Absolute	0.422	−0.366 to 1.211	−4.721	−6.078 to −3.364	5.565	4.208 to 6.923
	%	−0.299	−0.734 to 1.331	−6.439	−8.217 to −4.661	7.036	5.258 to 8.814
Sodium	Absolute	−0.569	−1.192 to 0.055	−4.777	−5.850 to −3.704	3.639	2.557 to 4.713
	%	−0.407	−0.858 to 0.043	−3.447	−4.223 to −2.672	2.638	1.858 to 3.408
Potassium	Absolute	−0.032	−0.061 to 0.002	−0.222	−0.272 to −0.171	0.158	0.109 to 0.209
	%	1.034	0.209 to 1.860	−6.413	−7.843 to −4.999	4.353	2.931 to 5.775
Chloride	Absolute	2.323	−2.760 to −1.885	−5.143	−5.897 to −4.390	0.498	−0.256 to 1.251
	%	−2.188	−2.604 to −1.772	−4.873	−5.590 to −4.156	0.497	−0.220 to 1.214
Ionised calcium	Absolute	−0.009	0.017 to −0.013	−0.063	−0.0774 to −0.049	0.044	0.029 to 0.058
	%	−0.9736	−1.730 to −0.209	−5.959	−7.275 to −4.643	4.012	2.696 to 5.327
Glucose	Absolute	−0.062	−0.151 to 0.027	−0.643	−0.796 to −0.489	−0.519	0.365 to 0.672
	%	−2.4773	−5.39 to 0.445	−21.544	−26.576 to −16.512	16.589	11.557 to 21.621
Lactate	Absolute	−0.133	0.226 to −0.041	−0.739	−0.899 to −0.579	−14.439	7.667 to −11.212
	%	−2.209	−4.084 to −0.335	0.472	0.3124 to 0.632	10.021	6.793 to 13.248
Urea	Absolute	0.3561	0.128 to 0.584	−1.058	−1.451 to −0.666	1.771	1.378 to 2.163
	%	2.537	−0.157 to 5.23	−14.195	−18.838 to −9.552	19.269	14.626 to 23.912
Creatinine	Absolute	12.511	2.308 to 22.714	−54.052	−71.619 to −36.485	79.075	61.507 to 96.642
	%	4.564	0.329 to 8.798	−23.064	−30.355 to −15.772	32.191	24.899 to 39.482

CI, confidence interval; pCO_2_, partial pressure of carbon dioxide; pO_2_, partial pressure of oxygen.

The SPP+ urea and creatinine showed acceptable analytical agreement with the Roche cobas c702. Even though the bias noted with urea was within APS, we noted a concentration-dependent bias (Figure 1). The SPP+ had a negative bias at lower concentrations (< 5 mmol/L) and a positive bias to the Roche cobas c702 at higher urea concentrations (> 10 mmol/L).

#### Linearity

All analytes showed acceptable linearity except urea. Urea did not meet APS at the lowest concentration level (2.3 mmol/L) in external quality assessment material. It exceeded the allowable non-linearity percentage of 13.3% and had a deviation of −12.20%. Despite not meeting APS, the urea concentration at the lower level was within external quality assessment APS and was therefore deemed acceptable.

### Clinical validation

Fifty-one (51) patients were recruited for estimation of dialysis adequacy and C_index_ by measuring urea and creatinine in pre- and post-haemodialysis samples (Online Supplementary Figure 1). Collection was completed for 35 haemodialysed patients: 16 women, 19 men; median age was 42.5 years (interquartile range 32–51 years). All data were not normally distributed. Post-dialysis weight had a median of 66.0 kg (interquartile range 56.5 – 72.5 kg) for women and 64.0 kg (interquartile range 53.5 – 70.8 kg) for men.

#### Haemodialysis adequacy by SpKt/V and urea reduction ratio

The median of SpKt/V for both the SPP+ and Roche cobas c702 analysers was 1.5 (*p* = 0.626) (Table 4). The median URR for SPP+ was 71.3 and for Roche cobas c702, 71.5 (*p* = 0.844). When classifying the minimum SpKt/V, there was 88.6% agreement between the two analysers, with a Cohen’s kappa statistic of 0.44 (Table 5). For classification of optimal SpKt/V, the agreement was 77.1%, with a Cohen’s kappa statistic of 0.47.

**TABLE 4:** Comparison of Roche cobas c702 and Stat Profile Prime Plus systems for haemodialysis adequacy assessment and creatinine index measurement, Johannesburg, South Africa, June 2023 to December 2023.

Parameter	Roche cobas c702 (*n* = 35)		Stat Profile Prime Plus (*n* = 35)		*p*
	Median	IQR	Median	IQR	
Single-pool Kt/V	1.5	1.3–1.8	1.5	1.3–1.7	0.626
Urea reduction ratio	71.50	67.58–76.95	71.30	66.95–76.00	0.844
Minimum creatinine index	22.78	20.76–23.74	19.63	18.70–21.32	< 0.0001

IQR, interquartile range.

**TABLE 5:** Clinical agreement between Roche cobas c702 and Stat Profile Prime Plus for assessing minimum and optimal single pool Kt/V and creatinine index in haemodialysis, Johannesburg, South Africa, June 2023 to December 2023.

Variable	Total agreement	Cohen’s Kappa	95% CI
Minimum SpKt/V	88.6	0.44	−0.01–0.89
Optimal SpKt/V	77.1	0.47	0.16–0.78
Minimum creatinine index	34.3	0.24	0.05–0.41

CI, confidence interval; SpKt/V, single-pool Kt/V.

#### Protein-energy wasting by creatinine index

In evaluating C_index_, the Roche cobas c702 analyser showed a median C_index_ of 22.78 (Table 4), whereas the SPP+ analyser had a median C_index_ of 19.63 (*p* < 0.0001). For the minimum C_index_, agreement between the two analysers was 34.3%, with a Cohen’s kappa of 0.24 (Table 5).

## Discussion

The SPP+ demonstrated acceptable precision, with performance comparable to central laboratory analysers for blood gases, electrolytes, glucose and lactate. It showed good agreement in haemodialysis adequacy assessment, supporting the potential of POCT in decentralising haemodialysis monitoring. However, a proportional bias was observed in creatinine measurements (4.56%), with poor agreement for C_index_ (34.30%) which may impact the assessment of PEW.

This study shows that SPP+ is fit for purpose with acceptable performance on analytical verification.^7^ The slightly lower correlation in sodium measurements aligns with findings from a 2020 South African study (*r* = 0.898), which also reported a weaker correlation between the Radiometer ABL800 FLEX^®^ (Radiometer, Copenhagen, Denmark) and SPP+.^7^ However, since correlation does not assess agreement between methods, further assessment of bias (−0.41%) confirmed that performance of the SPP+ is within allowable performance limits and therefore the lower correlation was not clinically significant.

The SPP+ device shows good agreement in assessing haemodialysis adequacy, suggesting potential to expand POCT applications and improve clinical decision-making. The study found no statistically significant median differences in the measurement of SpKt/V and URR between the SPP+ and Roche cobas c702 analysers (*p* = 0.626 and *p* = 0.844, respectively), supporting the utility of the SPP+ for haemodialysis adequacy.

A good agreement was seen in haemodialysis adequacy assessment for minimum (agreement = 88.6% and Cohen’s kappa = 0.44) and optimal SpKt/V (agreement = 77.1% and Cohen’s kappa = 0.47) and by URR (agreement = 88.6% and Cohen’s kappa = 0.44). Despite the low Cohen’s kappa values, which typically indicate weak agreement, the high percentage agreement suggests that the SPP+ and central laboratory analyser still show acceptable concordance in assessing haemodialysis adequacy. The discrepancy in kappa values is likely because of the non-parametric distribution of data, which can introduce bias and inflate the likelihood of chance agreement. Our findings align closely with those of a 2021 study in France that demonstrated that POCT haemodialysis assessment show acceptable agreement with the Roche cobas c701 analyser and an ultra-performance liquid chromatography tandem mass spectrometry method.^22^ However, in this French study, acceptable agreement was also found for creatinine measurement and C_index_ using the ABL90 FLEX PLUS® blood gas analyzer (Radiometer Medical ApS, Copenhagen, Denmark). analyser. In our study, we observed a proportional bias of serum creatinine (bias 4.56% [0.33–8.80]), especially evident at higher concentrations. In comparison with Roche cobas c702, SPP+ had a constant systematic error, with a *y*-intercept of 15.04. Although, we only report a comparison with one analyser, our findings were consistent with studies in Guatemala and the United Kingdom, where POCT creatinine inaccuracies at high concentrations skewed clinical interpretations and required a correction factor.^24^

In our study, creatinine index (C_index_) was used as a surrogate for PEW. Low C_index_ has been found to be associated with poor cardiovascular outcomes. Our findings on the C_index_ showed statistically significant differences (*p* < 0.0001) and poor agreement between the SPP+ and Roche cobas c702 analysers (agreement = 34.3 %, Cohen’s kappa of 0.24). The suboptimal agreement between the analysers could be attributed to reduced linearity at higher creatinine concentrations. Our study findings parallel those of a 2023 study in Nicaragua and Guatemala that demonstrated a rise in the inaccuracy with increasing concentrations of creatinine.^10,24^ A second study conducted in Guatemala in 2017 also showed that POCT creatinine consistently overestimated the creatinine by an average of 22% and the disagreement appeared greater at higher concentrations of serum creatinine.^24^

The SPP+ analyser shows promise for decentralising POCT in haemodialysis, its performance in measuring creatinine and the C_index_ raises concerns, particularly in patients with high serum creatinine concentrations. The observed proportional bias in creatinine measurements and poor agreement in C_index_ could significantly impact clinical decisions related to PEW and cardiovascular risk. Given the increasing prevalence of end-stage kidney disease in regions with limited resources such as South Africa, further research is necessary to refine POCT technologies for biomarkers such as creatinine, to ensure their accuracy at high concentrations. Future studies should explore broader analyser comparisons and clinical validation in more populations with advanced CKD to improve the reliability of these devices in clinical practice.

### Strengths

The strengths of our study include incorporation of established CLSI guidelines adding to the quality and validity of the study. Furthermore, the study evaluated the clinical utility of the SPP+ analyser in haemodialysis. This clinical validation adds practical relevance to this evaluation and highlights the potential impact on patient care.

### Limitations

The method comparison was restricted to remnant patient samples, which did not cover the entire analytical range. Despite being adequately powered (> 80%), the sample size of patients attending the haemodialysis unit remains relatively small. Furthermore, the performance of the SPP+ was only compared to two other analysers in our study. This could affect the extent to which our findings can be generalised beyond this specific patient population and set of analysers. Finally, pre-analytical factors and implementation costs were not assessed in our study.

### Conclusion

As CKD and treated kidney failure become more common, our study supports the use of POCT to assess acid-base status, electrolyte balance, metabolic parameters, and dialysis adequacy. However, it should not replace central laboratory testing for creatinine measurement in patients on chronic haemodialysis. We recommend using central laboratory analysis for this purpose.

## References

[1] Vashist SK. Point-of-care diagnostics: Recent advances and trends. Biosensors. 2017;7(4):62. 10.3390/bios7040062 29258285 PMC5746785

[2] Khan AI, Pratumvinit B, Jacobs E, et al. Point-of-care testing performed by healthcare professionals outside the hospital setting: Consensus-based recommendations from the IFCC Committee on Point-of-Care Testing (IFCC C-POCT). Clin Chem Lab Med. 2023;61(1):5–12. 10.1515/cclm-2023-0502 37267483

[3] Khan AH, Shakeel S, Hooda K, Siddiqui K, Jafri L. Best practices in the implementation of a point of care testing program: Experience from a tertiary care hospital in a developing country. J Clin Lab Anal. 2019;33(5):e22853. 31695586 PMC6803771

[4] Alexandra S. Changes in the new ISO 15189:2022. Washington, DC: ANSI National Accreditation Board; 2022.

[5] Trenti T. Synergy between point-of-care testing and laboratory consolidations. Clin Chem Lab Med. 2021;59(8):1285–1291. PMC8592627 34819822

[6] Liguori G, Belfiore P, D’Amora M, Liguori R, Plebani M. The principles of health technology assessment in laboratory medicine. Clin Chem Lab Med. 2017;55(1):32–37. 10.1515/cclm-2016-0371 27341564

[7] Jawa A, Motara F, Moolla M, Laher AE. A comparative assessment of the Nova STAT Profile Prime Plus^®^ critical care analyzer. Cureus. 2020;12(8):e10015. 10.7759/cureus.9932 32968593 PMC7505621

[8] Bogaert L, Schiemsky T, Van Hover P, De Schrijver P, Van Hoovels L. Analytical and diagnostic performance evaluation of five creatinine POCT devices in the identification of patients at risk for post-contrast acute kidney injury (PCAKI). Clin Chem Lab Med. 2019;57(9):e214–e217. 10.1515/cclm-2018-1105 30710476

[9] Lee HY, Ahn S, Kim H, Lee W, Chun S, Min WK. Performance evaluation of the Stat Profile pHOx Ultra blood gas analyzer. J Lab Med Qual Assur. 2019;41(1):47–49. 10.15263/jlmqa.2019.41.1.47

[10] Dally M, Amador JJ, Butler-Dawson J, et al. Point-of-care testing in chronic kidney disease of non-traditional origin: Considerations for clinical, epidemiological, and health surveillance research and practice. Ann Glob Health. 2023;89(1):15. 10.5334/aogh.3884 36789382 PMC9896998

[11] Daugirdas JT, Depner TA, Inrig J, et al. KDOQI clinical practice guideline for hemodialysis adequacy: 2015 update. Am J Kidney Dis. 2015;66(5):884–930. 10.1053/j.ajkd.2015.07.015 26498416

[12] Ikizler TA, Burrowes JD, Byham-Gray LD, et al. KDOQI clinical practice guideline for nutrition in CKD: 2020 update. Am J Kidney Dis. 2020;76(3 suppl 1):S1–S107. 10.1053/j.ajkd.2020.05.006 32829751

[13] Paget G, Naicker S, Assounga A, et al. Guideline for the optimal care of patients on chronic dialysis in South Africa. S Afr Med J. 2015;105(5):401–405.

[14] Birajdar N, Anandh U, Premlatha S, Rajeshwari G. Hand grip strength in patients on maintenance hemodialysis: An observational cohort study from India. Indian J Nephrol. 2019;29(6):393–397. 10.4103/ijn.IJN_251_18 31798220 PMC6883853

[15] Carrero JJ, Stenvinkel P, Cuppari L, et al. Etiology of the protein-energy wasting syndrome in chronic kidney disease: A consensus statement from the International Society of Renal Nutrition and Metabolism (ISRNM). J Ren Nutr. 2013;23(2):77–90. 10.1053/j.jrn.2013.01.001 23428357

[16] Bereket TL, Anuja S, Joel DK. Is it important to prevent and treat protein-energy wasting in chronic kidney disease and chronic dialysis patients?. J Ren Nutr. 2018;28(6):369–379. 10.1053/j.jrn.2018.04.002 30057212

[17] Canaud B. Simplified creatinine index as a new tool for monitoring protein energy malnutrition and predict outcome risk in hemodialysis patients: Recent findings and perspectives. Nephrol Renal Ther. 2021;7(2):1–5. 10.24966/NRT-7313/100050

[18] Engel N, Yellappa V, Davids M, Dheda K, Pai NP, Pai M. Barriers to point of care testing in India and South Africa. In: Hostettler S, Hazboun E, Bolay JC, editors. Technologies for development. Cham: Springer International Publishing, 2018; p. 75–85.

[19] Cohen GM, Drain PK, Noubary F, Cloete C, Bassett IV. Diagnostic delays and clinical decision making with centralized Xpert MTB/RIF testing in Durban, South Africa. J Acquir Immune Defic Syndr. 2014;67(3):e88–e93. 10.1097/QAI.0000000000000309 25314255 PMC4197409

[20] Bargnoux AS, Kuster N, Sutra T, et al. Evaluation of a new point-of-care testing for creatinine and urea measurement. Scand J Clin Lab Invest. 2021;81(4):290–297. 10.1080/00365513.2021.1914344 33908840

[21] Clinical and Laboratory Standards Institute (CLSI). User verification of precision and estimation of bias; EP15-A3. 3rd ed. Wayne (PA): CLSI; 2014.

[22] Clinical and Laboratory Standards Institute (CLSI). Evaluation of the linearity of quantitative measurement procedures: A statistical approach; approved guideline. 2nd ed. Wayne (PA): CLSI; 2020.

[23] Clinical and Laboratory Standards Institute (CLSI). Measurement procedure comparison and bias estimation using patient samples; approved guideline. 3rd ed. Wayne (PA): CLSI; 2018.

[24] Griffin BR, Butler-Dawson J, Dally M, et al. Unadjusted point-of-care creatinine results overestimate acute kidney injury incidence during field testing in Guatemala. PLoS One. 2018;13(9):e0204614. 10.1371/journal.pone.0204614 30261074 PMC6160126

